# Development and Evaluation of a Molecular Test for Monkeypox Virus in the Federal District, Brazil

**DOI:** 10.3390/genes16070779

**Published:** 2025-06-30

**Authors:** Lucas Pereira da Silva, Fabián Andrés Hurtado, Aline Belmok, Rafael Correa, Claudia F. Sousa, Gislene P. Gil, Lara Velasco, Rafael H. Jácomo, Lídia F. Nery, Maria Tereza de Oliveira Rodrigues, Miguel S. Andrade, Rosângela Vieira de Andrade

**Affiliations:** 1 Molecular Biology Division, Sabin Diagnóstico e Saúde, Brasília 70632-300, DF, Brazil; 2 Graduate Program in Genomic Sciences and Biotechnology, Catholic University of Brasília, Brasília 71966-700, DF, Brazil

**Keywords:** monkeypox virus (MPXV), qPCR, molecular detection, F3L

## Abstract

Background: Monkeypox virus, the etiological agent of Mpox, is a double-stranded DNA virus belonging to the Orthopoxvirus genus that has attracted increasing attention due to sporadic outbreaks in humans. In 2022, it was responsible for the largest Mpox outbreak outside the African continent, infecting over 117,000 individuals worldwide. In Brazil, since the first confirmed case in June 2022, more than 13,000 people have been diagnosed with the virus. Methods: In July 2022, we developed the first molecular test for the detection of monkeypox virus in the Midwest region of the country, allowing the diagnosis of the disease in various patients, mainly residents of the Federal District. Thus, in this work, we present the validation of a laboratory-developed qPCR test (LDT) for monkeypox virus detection, as well as a retrospective epidemiological analysis based on laboratory results. Results: The developed qPCR test demonstrated 100% accuracy, with a detection limit of 21.25 copies per reaction, and was validated for samples from swabbed pustule exudates and lesion crusts. To date, 295 tests have been conducted, with 88 (30%) returning positive. The positivity rate was 41.15% in male patients and 2.41% in female patients. A peak in positivity was observed in August 2022. From 2023 to 2024, there was a marked decline in test demand with occasional positive results. Conclusions: The rapid implementation of the test by our laboratory allowed for an immediate response to patients and provided important data for understanding the dynamics of monkeypox virus spread in Brazil, particularly in the Midwest region.

## 1. Introduction

Mpox, a disease for which the etiological agent was identified in 1958 in Denmark, is caused by a double-stranded DNA virus of the Orthopoxvirus genus [1,2,3]. The first reported outbreaks of Mpox occurred in Central and West Africa, dating back to the 1970s, with symptoms such as headache, fever, lymphadenopathy, and systemic epithelial lesions characterized by pustules similar to human smallpox. The virus replicates in the dermal cells and spreads via the lymph nodes, potentially affecting other organs. Infected individuals remain contagious until the lesions have completely healed, with recovery usually occurring in 4 to 5 weeks [4,5,6]. Current cases constitute the largest and most widespread non-endemic Mpox outbreak known to date and were declared a public health emergency of international concern by the World Health Organization (WHO) in July 2022 [7,8,9].

In June 2022, the first case of Mpox in Brazil was detected in a 41-year-old resident of São Paulo who had traveled to Portugal and Spain and reported sexual contact with three different individuals [10]. From this case in June 2022 until 31 December 2024, 13,429 cases were confirmed in Brazil, with the highest incidence in the Midwest and Southeast regions of the country [11].

Considering the importance of early and accurate detection of the virus for its containment and for implementation of public health measures, we developed the first molecular test for the detection of monkeypox virus in the Midwest region of Brazil in July 2022, enabling the diagnosis of the disease in a range of patients, mostly residing in the Federal District (DF), which includes Brasilia, the capital of Brazil. In this paper, we present the validation of a laboratory-developed qPCR test (LDT) for monkeypox virus detection and conduct a retrospective epidemiological analysis based on laboratory results.

## 2. Materials and Methods

### 2.1. Samples

For assay validation, swab samples from skin lesions resembling Mpox-associated pustules were spiked with viral material obtained from the first confirmed monkeypox case in Brazil [12]. An aliquot of the virus, previously isolated and cultured in Vero CCL-81 cells (ATCC^®^ CCL-81™), was kindly provided by the Institute of Tropical Medicine at the University of São Paulo (USP). Although the exact viral titer of this material was not determined, its use aimed to simulate positive samples for assay validation. The samples were collected using Rayon swabs and stored in digene^®^ HC2 DNA Collection Devices (Qiagen, Hilden, Germany).

### 2.2. DNA Extraction

The assay was initially validated using the QiaSymphony system (Qiagen, Hilden, Germany) with the DSP Virus/Pathogen Mini and Midi kits. For this purpose, spiked samples were used, consisting of inactivated monkeypox virus particles added to negative clinical swab matrices. The QIA Symphony system (Qiagen, Hilden, Germany) offers two options for primary sample input volume—200 µL (Mini kit) and 400 µL (Midi kit)—as well as three options for nucleic acid elution volume: 60 µL, 85 µL, and 110 µL. Based on comparative testing, the combination of a 400 µL sample input with a 60 µL elution volume using the DSP Virus/Pathogen Midi Kit was selected as the standard protocol. Nucleic acid extraction was subsequently validated on the Maxwell RSC platform using the Maxwell RSC Total Viral Nucleic Acid Kit (Promega, Madison, WI, USA), following the manufacturer’s instructions, with 300 µL of primary sample input and a 60 µL elution volume.

### 2.3. Real Time PCR

The primers and probe for real-time PCR (qPCR) were selected from a conserved region of the open reading frame F3L, as described by Maksyutov et al. (2016) [12], and synthesized by Integrated DNA Technologies (IDT, Coralville, IA, USA). The human RNase P gene was chosen as the target for endogenous control. A synthetic oligonucleotide containing the target sequence for the monkeypox primers, along with 10 base pairs upstream and 11 base pairs downstream of the amplified region, was used as a positive control.

The amplification reactions were performed in triplicate using the LightCycler Multiplex DNA Master Kit (Roche Diagnostics, Mannheim, Germany), and amplification was carried out on the LightCycler 480 II, following the manufacturer’s recommendation. The qPCR reactions were performed using the following thermal cycling conditions: an initial denaturation step at 95 °C for 10 min, followed by 40 amplification cycles consisting of 95 °C for 30 s (denaturation) and 60 °C for 30 s (annealing and extension). Fluorescence signal acquisition was carried out at the end of each 60 °C step. The threshold cycle (Ct) cutoff for calling a sample positive was set at the final cycle of the reaction; thus, only amplification curves with Ct values below 40 were considered positive.

The results were deemed acceptable when the Ct value for human RNase P was below 35. All experiments included a non-template control (NTC) and a negative control (NC) to verify the specificity of the reaction.

### 2.4. Sanger Sequencing

**Figure 1:**
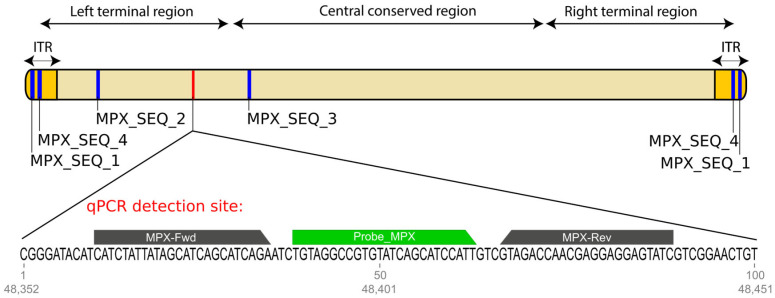
Schematic representation of the monkeypox virus genome showing the qPCR detection target (in red) and the regions selected for sequencing (in blue). The sequence corresponding to the qPCR detection site is highlighted, with the primers and probe indicated.

To confirm the initial positive results, we performed Sanger sequencing of four regions of the monkeypox genome (Figure 1). The oligonucleotides used for amplification and sequencing were designed in this study using Geneious software (version 2022.0.2, Biomatters, Auckland, New Zealand) and are listed in Table 1. Details of the sequencing methodology can be found in Section 2 of the Supporting Information.

### 2.5. Limit of Detection (LoD)

To determine the Limit of Detection (LoD), an initial quantification step was performed using a serial dilution of a synthetic oligonucleotide containing the MPXV target sequence. This dilution series was used to generate a standard curve (Ct vs. copies/µL), which enabled the estimation of the concentration (copies/µL) of the eluate obtained from a spiked sample. The Ct value of the eluate was interpolated on this curve to calculate the corresponding copy number. Since 5 µL of template was used per reaction, the concentration in copies/µL was converted to copies per reaction by multiplying the value by 5.

Then, the quantified spiked sample eluate was serially diluted and tested in replicate reactions to assess detection performance at low concentrations. The number of replicates varied according to the dilution level, as shown in Section 3.3. Specifically, dilution points corresponding to concentrations up to 463.23 copies per reaction were tested in triplicate (n = 3). The remaining points, including the final 1:10 dilution and the three subsequent 1:2 serial dilutions, were tested in 21 replicates each (n = 21). The LoD was estimated using Probit regression analysis, based on the proportion of positive results observed at each dilution level.

The final LoD was calculated using the following equation:

$$LoD=\frac{{5+\Phi }^{-1}(0.95)-a}{b}$$

where $\Phi^{-1}(0.95)$ represents the inverse of the cumulative distribution function (CDF) of the standard normal distribution evaluated at 0.95 (i.e., the 95th percentile), which equals approximately 1.64485. In this equation, *a* is the intercept, and *b* is the slope coefficient of the Probit regression curve obtained from the experimental data.

### 2.6. Specificity

To assess the specificity of the qPCR assay, the primers were analyzed using the on line version of Primer-BLAST available at https://www.ncbi.nlm.nih.gov/tools/primer-blast/ (accessed on 1 June 2022) and Geneious Prime (version 2022.0.2, Biomatters, Auckland, New Zealand). Experimental specificity was further evaluated by processing clinical samples known to be positive for other pathogens, including *Chlamydia trachomatis*, *Neisseria gonorrhoeae*, varicella-zoster virus, and SARS-CoV-2, using the complete workflow validated for monkeypox virus detection—from nucleic acid extraction to specific qPCR amplification—to confirm the absence of non-specific amplification.

### 2.7. Analytical Precision and Reproducibility

To assess analytical precision, three samples with known results (one positive and two negatives) were processed and analyzed in triplicate across three different experiments on separate days (reproducibility) and in triplicate on the same day from extraction (repeatability).

### 2.8. Epidemiological Data and Analysis

Data from molecular tests performed using this methodology between July 2022 and December 2024 were utilized in a retrospective cross-sectional study. A total of 295 results, from 290 patients, were selected for epidemiological analysis. The dataset includes collection date, collection location (state and city), age, sex, and monkeypox qPCR assay results. Personal data were anonymized in accordance with the laboratory’s internal data handling policy and in compliance with the General Data Protection Law (Federal Law No. 13,709, of 14 August 2018).

The epidemiological analysis aimed to evaluate the positivity profile of Mpox across various demographic and geographic factors. Positivity was analyzed by sex, comparing rates between male and female individuals to assess potential differences in susceptibility or exposure. Age groups were established to examine positivity across different age brackets, providing insights into the age distribution of positive cases. Geographic regions, as well as state and city levels, were used to identify regional trends or hotspots, enabling a localized analysis of the disease’s spread.

## 3. Results

### 3.1. DNA Extraction

To optimize DNA extraction protocols, qPCR reactions were performed on eluates obtained from spiked samples, prepared by adding inactivated monkeypox virus to negative clinical swab matrices. Eluates from the different extraction conditions were tested in triplicate qPCR reactions, and the resulting Ct values (Table 2) were compared. The DS Virus/Pathogen Midi protocol, using 400 µL of input and 60 µL of elution and yielding the lowest Ct value, was chosen as the standard protocol for QiaSymphony extraction. For Maxwell extraction, the use of 300 µL of input and 60 µL of elution yielded similar results.

### 3.2. qPCR Assay Standardization

**Figure 2:**
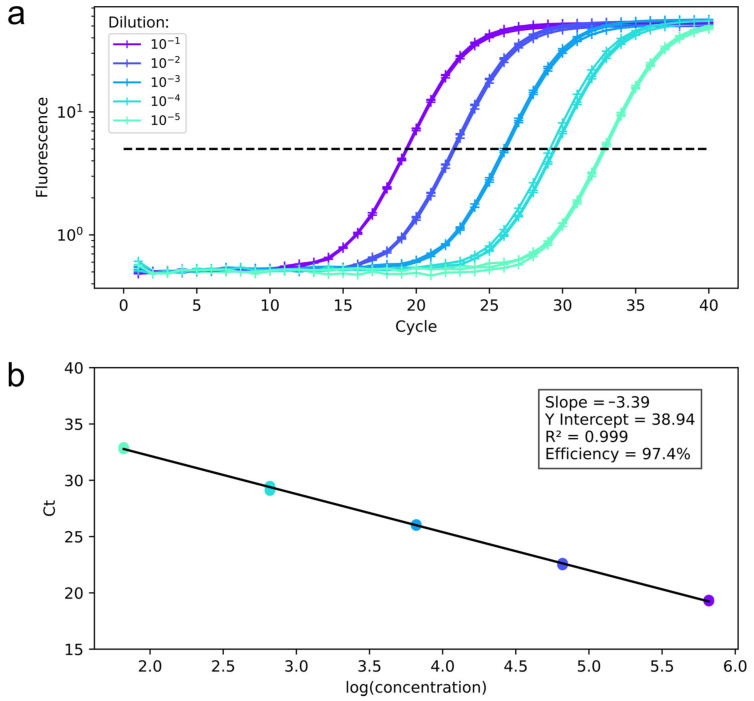
qPCR efficiency parameters for the MPXV target. (**a**) Amplification curves obtained from a ten-fold serial dilution of the standard template, The dashed line indicates the threshold; (**b**) Standard curve derived from the same dilution series. The calculated amplification efficiency was 97.4%, with a slope of −3.39.

The qPCR protocol was first standardized using an oligonucleotide containing the target monkeypox sequence (standard template) and spiked samples derived from virus culture. Serial dilutions of the standard template were used for qPCR reactions, and the resulting Ct values were plotted to construct a standard curve, which was then used to estimate the efficiency of the qPCR (Figure 2). The efficiency was 97.4%, with a slope of −3.39 and an R^2^ value of 0.999 for the MPXV target.

### 3.3. Sensitivity (Limit of Detection—LoD)

**Figure 3:**
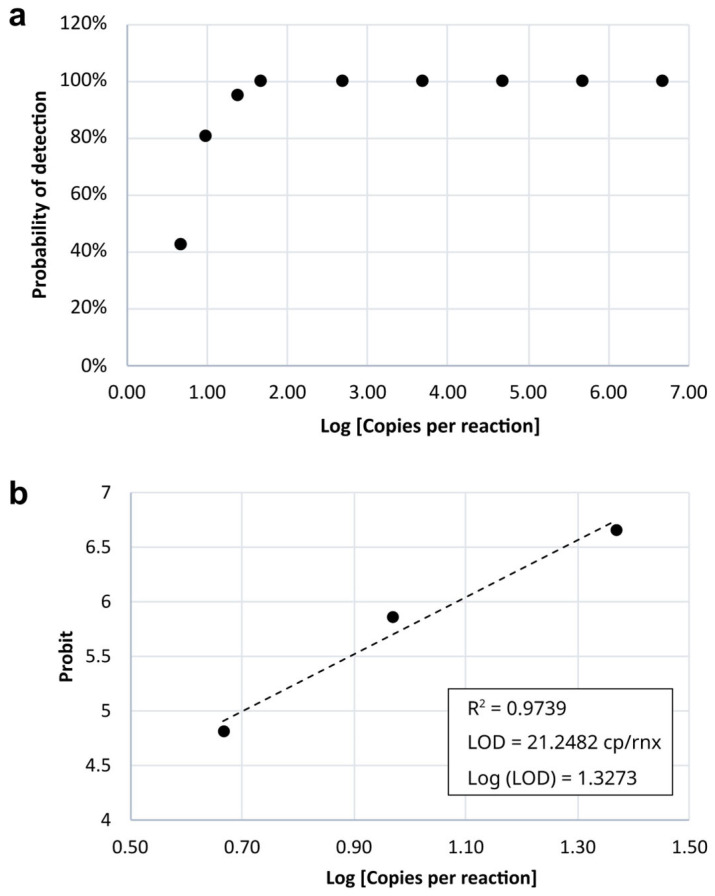
Limit of detection (LOD) analysis of the qPCR assay. (**a**) Probability of detection versus log₁₀ of target copies per reaction; (**b**) Probit regression to determine the LOD, defined as the number of copies per reaction with a 95% detection probability. The dashed line represents the best-fit regression line used to estimate the LOD. The LOD was calculated as 21.2482 copies/reaction, with a coefficient of determination (R^2^ = 0.9739).

The results of probit regression analysis for estimating the LoD across different dilution factors are shown in Table 3. Figure 3 illustrates the detection probability as a function of the number of DNA copies per reaction. The estimated LoD, with 95% confidence, was 21.25 copies per reaction.

### 3.4. Specificity

In the specificity test, none of the positive samples for SARS-CoV-2, Herpes simplex virus 1 (HSV-1), Hepeviridae (HEV), Roseolovirus (HHV-7), or varicella-zoster virus yielded a positive result for monkeypox virus. As expected, amplification was observed only in spiked samples, further supporting the *in silico* assay results regarding monkeypox virus primer specificity. The results are presented in Table S1.

### 3.5. Analytical Precision and Reproducibility

Repeatability (consistency of results within the same assay) and reproducibility (consistency of results across different assays) were evaluated, and the results demonstrated high consistency across all experiments conducted, with 100% concordance observed in all replicates.

### 3.6. Sanger Sequencing

The first positive sample was subjected to PCR amplification, targeting four regions across the monkeypox genome and sequenced by Sanger. The sequences confirmed the presence of a monkeypox genome in the sample. The obtained sequences showed 100% identity with the NCBI Reference Sequence (NC_003310). The sequencing results are presented in Figure S1.

### 3.7. Accuracy

The samples from the first 30 patients, collected between 11 July 2022 and 4 August 2022, were also tested at LACEN-DF, the reference laboratory of the public healthcare network in the Federal District. The comparison of results demonstrated 100% concordance, as shown in Table S2.

### 3.8. Monkeypox Ct Values

**Figure 4:**
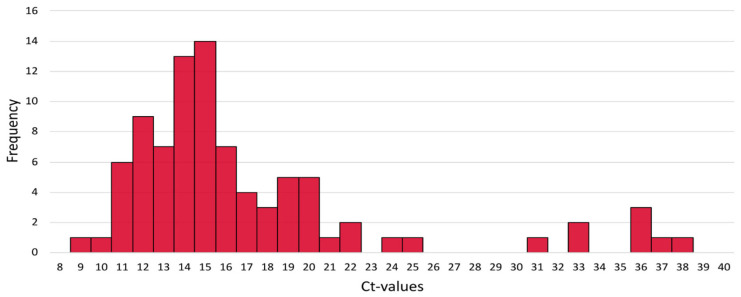
Distribution of Ct means of all monkeypox virus detections: the X-axis expresses the Ct value and the Y-axis the frequency of the results obtained.

To assess the distribution of Ct values for positive test results, mean Ct values across replicates for each sample (n = 88) were plotted (Figure 4). The average Ct value for the monkeypox target was 16.62, with a range from 8.83 to 37.37, and the mode was 18.73.

### 3.9. Spatial and Temporal Distribution of Monkeypox Detection

**Figure 5:**
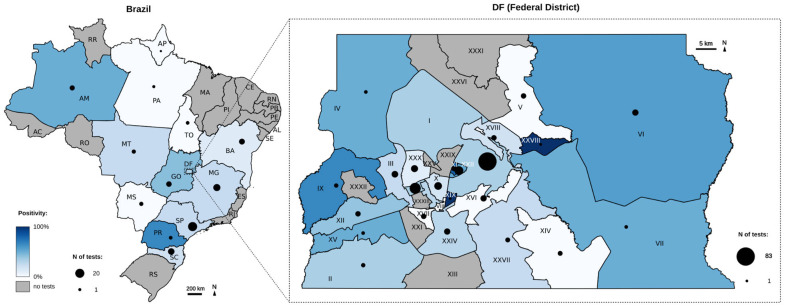
Mpox cases in Brazil and the Federal District detected by our test from July 2022 to December 2024: geographic analysis showing the total number of cases (black dots) in relation to the positive results in relation to the total (blue). Identification of the regions of the Federal District is presented in the Supplementary Materials, Table S4.

The 295 samples from 290 patients analyzed in this study originated from 12 Brazilian states (n = 81) and the Federal District (n = 214). The geographic distribution of the number of tests and positivity rates across Brazil is presented as a heat map in Figure 5. The highlighted area corresponds to the Federal District (DF), with the boundaries of its satellite cities delineated. Most of the tests were performed in Plano Piloto (n = 83), Águas Claras (n = 22), and Sudoeste (n = 16), with positivity rates of 30.12% (25/83), 56.25% (9/16), and 31.81% (7/22), respectively. A comprehensive dataset is available in Tables S3 and S4.

**Figure 6:**
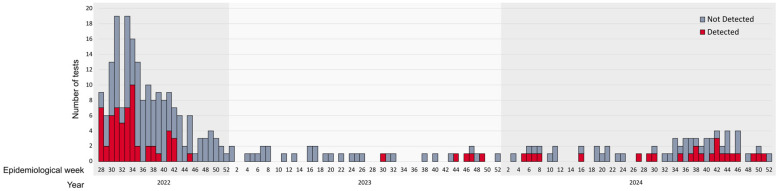
Weekly epidemiological assessment of monkeypox virus (MPXV) detection. Bars represent the total number of tests performed per week; positive results are shown in red and negative results in gray.

The temporal distribution of tests and positivity rates over the years 2022, 2023, and 2024 are presented in Figure 6. The period immediately following the virus’ introduction in Brazil was characterized by a sharp increase in both the number of tests performed and the number of positive results, followed by a subsequent decline. In 2023, testing activity was minimal. In 2024, a slight increase in both positivity rates and the number of tests conducted was observed during the second half of the year. Positivity rates in the DF were 36% (49/135) in 2022, followed by 16% (5/30) in 2023 and 26% (13/49) in 2024. In other states, positivity rates were 18% (10/57) in 2022, 0% (0/4) in 2023, and 58% (11/19) in 2024. The average turnaround time (TAT) was 2 days for samples from the DF and 3 days for samples from other states.

### 3.10. Demographic Characteristics

**Figure 7:**
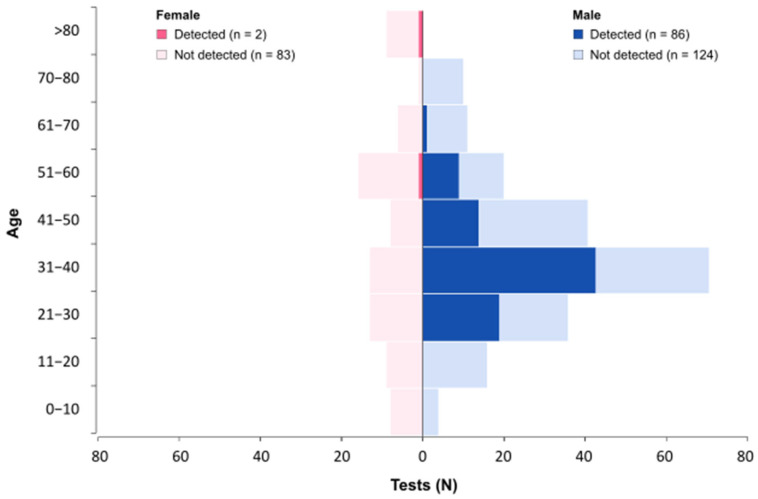
Age distribution of identified monkeypox virus (MPXV) cases. Bars are color-coded by sex (blue for males and pink for females). Darker shades indicate the number of positive results, while lighter shades represent negative results within each age group.

Of the 290 patients, 207 were male and 83 were female. Most of the positive results, 97.72% (86/88), are from samples obtained from male patients. The average age was 39 years for males (ranging from 2 to 77 years) and 42 years for females (ranging from 11 months to 103 years). The distribution of tests performed and positivity rates across age groups is presented in Figure 7. Among males, 50% (n = 43) of all positive results were observed in the 31 to 40 age group. Among females, no dominant age group was identified, with only two positive cases detected, one in the 51 to 60 group and another in the >80 group.

## 4. Discussion

This study presents the validation data of the molecular test that enabled the first diagnosis of an Mpox case in the Federal District of Brazil, as well as the epidemiological data of the diagnoses performed using this method. The test was developed shortly after the first case was detected in Brazil, in the state of São Paulo [10,14]. Using artificially contaminated (spike-in) samples with the first Brazilian isolate, we validated the method, which includes nucleic acid extraction and qPCR detection. Considering the absence of positive samples during the initial test validation, Sanger sequencing of four regions of the viral genome was used to confirm the first positive results in patient samples. To ensure the reliability of the method, its accuracy was determined through a comparative analysis with the reference laboratory (LACEN-DF), as detailed below.

The method innovatively combines sample collection, transport medium, and qPCR amplification for virus detection. The selected collection and transport medium is typically used for HPV testing, and to the best of our knowledge, there are no previous reports of its use for Mpox diagnosis. The chosen primers are specific for monkeypox virus clades 1 and 2 and do not show cross-reactivity with other Orthopoxviruses [12]. The analysis of the obtained Ct values demonstrates that the method can recover a high number of viral DNA copies from pustules and lesion crusts collected with swabs and transported in digene^®^ HC2 DNA Collection Devices (Qiagen, Hilden, Germany), as evidenced by the low Ct values shown in Figure 4. This finding is consistent with the high viral load observed in Mpox-associated lesions [15,16,17]. Further studies are needed to compare this medium’s performance with others and assess viral DNA stability for long-term storage and transport.

The validation results of our qPCR assay align with previous studies, with a limit of detection (LoD) of 21.25 copies per reaction, consistent with similar assays reporting LoDs ranging from 1 to 40 copies per reaction [15,18,19,20]. Our assay demonstrated 97.4% efficiency, within the recommended range for qPCR assay reproducibility and accuracy, showing 100% concordance with LACEN-DF and thus highlighting its robustness. For this comparison, the first 30 clinical samples tested in our laboratory were also analyzed at LACEN-DF using the CDC’s monkeypox-specific primer–probe set and the same qPCR platform (LightCycler^®^ 480 II, Roche Diagnostics, Rotkreuz, Switzerland). LACEN-DF is one of the Ministry of Health-designated laboratories responsible for evaluating molecular assays for monkeypox virus. Although our assay was already in clinical use, the 100% concordance observed in the comparative analysis led to its formal recognition by LACEN-DF. As a result, diagnostic reports issued using our method were accepted as valid for inclusion in the official national surveillance statistics. These findings confirm the reliability of our assay for Mpox detection and its potential for epidemiological surveillance, with our lab maintaining 100% performance indicators in an external quality control program from August 2022 to December 2024.

The positivity rate observed in the male group was 40.95%, while the rate in the female group was 2.35%. This finding is in accordance with previous studies [21,22] and official data from Ministry of Health [11]. As noted by Pascom et al. (2022) [23], although Mpox is not a sexually transmitted infection, the outbreak in Brazil and globally predominantly affected young males, particularly men who have sex with men (MSM), which is consistent with our data [23,24].

The highest viral dissemination periods coincided with the period in which we conducted most of the tests and observed the highest positivity rates, such as after the virus entered Brazil (epidemiological weeks 28–42) and at the end of 2024 (weeks 34–46). This trend mirrors official data from the Brazilian Ministry of Health [11]. As of 30 January 2024, 20% of reported cases were confirmed, and our data showed a 28.69% positivity rate. The DF had the highest national incidence, with 11 cases per 100,000 inhabitants, highlighting the need for testing in the region.

## 5. Conclusions

The standardization and implementation of the Mpox molecular test, a collaboration between Sabin Laboratory and the Catholic University of Brasília (UCB), enabled a rapid response to the outbreak in the Midwest region of Brazil. Its validation in 45 days highlights its effectiveness as a public health tool, ensuring fast and accurate identification of the virus. This test strengthens outbreak control and enhances preparedness for future public health challenges.

## Figures and Tables

**Table 1:** Oligonucleotide primers used in this study.

Name	Application	Sequence (5′-3′)	Reference
MPX-Fwd MPX-Rev Probe_MPX	qPCR	CATCTATTATAGCATCAGCATCAG	MAKSYUTOV, 2016 [12]
	qPCR	GATACTCCTCCTCGTTGGTCTAC	
	qPCR	/56-FAM/TGTAGGCCG/ZEN/TGTATCAGCATCCATT/3IABkFQ/	
RNase-P-Fwd RNase-P-Rev Probe_RNase-P	qPCR	AGATTTGGACCTGCGAGCG	adapted from Emery, et.al, 2004 [13]
	qPCR qPCR	GAGCGGCTGTCTCCACAA	
		/HEX/TTC TGA CCT/ZEN/GAA GGC TCT GCG CG-3IBkFQ	
Mpox_2139-F (MPX_SEQ_1) Mpox_2620-R (MPX_SEQ_1)	Sanger	GGATTCGCTGAGACCGGTAG	designed in this study
	Sanger	TATCGTGTCCTCCGGGAACT	designed in this study
Mpox_19,722-F (MPX_SEQ_2) Mpox_20,266-R (MPX_SEQ_2)	Sanger	TGGCAAATCTAACTGCGGGT	designed in this study
	Sanger	AATGACGCTATCCGACGGTC	designed in this study
Mpox_61,272-F (MPX_SEQ_3) Mpox_61,801-R (MPX_SEQ_3R)	Sanger	AGACCTATTCCCCCTGCCAT	designed in this study
	Sanger	TATGCCATTCTAGCCGCCAG	designed in this study
Mpox_193,245-F (MPX_SEQ_4) Mpox_193,792-R (MPX_SEQ_4)	Sanger	CCTCGTGTGGTGTATGCTCT	designed in this study
	Sanger	ACGTAGTGATCGTCGTAGGG	designed in this study

**Table 2:** Sample input and elution volume test for better reaction efficiency.

Kit	Sample Input	Elution	Average of Triplicates (Ct)	Minimum (Ct)	Maximum (Ct)
DSP virus/Pathogen MINI	200 µL	60 µL	18.66	18.49	18.88
		85 µL	19.34	18.94	19.91
		110 µL	19.12	18.94	19.39
DSP virus/Pathogen MIDI	400 µL	60 µL	17.54	17.34	17.91
		85 µL	18.08	17.82	18.7
		110 µL	18.22	18.07	18.4

**Table 3:** Probit regression results for estimating the limit of detection of different dilution factors.

Dilution Factor	Copies per Reaction	Log [Copies per Reaction]	Number of Replicates	Number of ‘Detected’ Results	Number of ‘Not Detected’ Results	Percentage of ‘Detected’ Results	Probit
1/10	4,632,308	6.67	3	3	0	100.00%	-
1/10	463,230.77	5.67	3	3	0	100.00%	-
1/10	46,323.08	4.67	3	3	0	100.00%	-
1/10	4632.31	3.67	3	3	0	100.00%	-
1/10	463.23	2.67	3	3	0	100.00%	-
1/10	46.32	1.67	21	21	0	100.00%	-
1/2	23.16	1.36	21	20	1	95.24%	6.67
1/2	9.26	0.97	21	17	4	80.95%	5.88
1/2	4.63	0.67	21	9	12	42.86%	4.82

## Data Availability

The original contributions presented in this study are included in the article/Supplementary Material. Further inquiries can be directed to the corresponding author(s).

## Supplementary Materials

The following supporting information can be downloaded at: https://www.mdpi.com/article/10.3390/genes16070779/s1, Table S1: Specificity test results table; Figure S1: The result of agarose gel electrophoresis and Sanger sequencing for sample 220322986300; Table S2: Comparability between our results and LACEN-DF results for 30 samples; Table S3: compiled from the data used in the study; Table S4: compiled from the data in Federal District, Brazil.

## Abbreviations

The following abbreviations are used in this manuscript:

MPOX	Re-emerging disease caused by the monkeypox virus
MPXV	Monkeypox virus
qPCR	Quantitative polymerase chain reaction
WHO	World Health Organization
DF	Federal District (Brazil)
NTC	Non-template control
NC	Negative control
Ct	Cycle threshold
LACEN-DF	Central Laboratory in the Federal District reference laboratory of the public Healthcare
MSM	Men who have sex with men
