# Supplementary material for: Development and Evaluation of a Molecular Test for Monkeypox Virus in the Federal District, Brazil
Source: Genes (Basel). 2025 Jun 30;16(7):779. doi: 10.3390/genes16070779 (PMC12294412; doi:10.3390/genes16070779)
Supplement: Supplementary file 1 [file genes-16-00779-s001.zip › Supplementary Material Table S1, Figure S1 and Table S2.pdf]

Supplementary material – Development and Evaluation of Rapid Diagnostic Tests for Monkeypox Virus in the Federal District, Brazil

1. Specificity

The specificity of the method was experimentally evaluated using a set of samples containing pathogens other than the Monkeypox virus. All samples were confirmed to be negative for Monkeypox. The results are summarized in **Table S1**, highlighting the method's ability to accurately distinguish the target virus from other pathogens.

Table S1. *Specificity Test Results Table. Samples positive for the pathogens SARS-CoV-2, Monkeypox, Varicella-Zoster virus (VZV), Herpes simplex virus 1 (HSV1), Human betaherpesvirus 7 (HHV7), and Hepatitis E virus (HEV) were used. The results were concordant with the expected outcome for all samples.*

Sample	Description	Previously identified pathogen	Expected result	Observed result	Concordance
Sample 01	Nasopharyngeal swab	SARS-CoV-2	Not detected	Not detected	Concordant
Sample 02	Culture supernatant from Monkeypox-infected Vero cells	Monkeypox	Detected	Detected	Concordant
Sample 03	Exudate from vesicular pustules	VZV,HHV7	Not detected	Not detected	Concordant
Sample 04	Exudate from vesicular pustules	VZV	Not detected	Not detected	Concordant
Sample 05	Exudate from vesicular pustules	VZV	Not detected	Not detected	Concordant
Sample 06	Exudate from vesicular pustules	VZV	Not detected	Not detected	Concordant
Sample 07	Exudate from vesicular pustules	VZV	Not detected	Not detected	Concordant
Sample 08	Exudate from vesicular pustules	VZV	Not detected	Not detected	Concordant
Sample 09	Exudate from vesicular pustules	VZV	Not detected	Not detected	Concordant
Sample 10	Exudate from vesicular pustules	VZV	Not detected	Not detected	Concordant
Sample 11	Exudate from vesicular pustules	VZV	Not detected	Not detected	Concordant
Sample 12	Exudate from vesicular pustules	HSV1,VZV	Not detected	Not detected	Concordant
Sample 13	Exudate from vesicular pustules	HSV1,HHV7	Not detected	Not detected	Concordant
Sample 14	Exudate from vesicular pustules	HSV1	Not detected	Not detected	Concordant
Sample 15	Exudate from vesicular pustules	HEV	Not detected	Not detected	Concordant

2. Sanger Sequencing

The DNA extracted from the first patient with a positive result was subjected to PCR amplification targeting four regions of approximately 500 base pairs (bp) across the Monkeypox genome, followed by Sanger sequencing to confirm the presence of the virus with a sequence compatible with Monkeypox.

Amplification was carried out using the QIAGEN Multiplex PCR Kit on a VeritiPro thermocycler (Thermo Fisher Scientific), following the protocol: 15 minutes at 95°C, followed by 35 cycles of 30 seconds at 94°C (denaturation), 90 seconds at 58°C (annealing) and 90 seconds at 72°C (extension). The success of amplification was confirmed by electrophoresis. After PCR amplification, the fragments were purified enzymatically using EXOSAP. Subsequently, the purified fragments underwent amplification with BigDye V3.1 cycle reagent (Thermofisher®) and were sequenced using the Applied Biosystems 3500 Genetic Analyzer according to the manufacturer's specifications. The resulting

sequences were analyzed using Geneious software and mapped against the reference sequence from the United States isolate (NC_003310).

Figure S1 (a) shows the result of agarose gel electrophoresis for sample 220322986300, with four distinct bands corresponding to the expected sizes. The sequence generated for the MPX_SEQ_3 region (see Figure 1 in the main text for details on the sequenced regions) is shown in **(b)**, where 100% identity can be observed in the two strands generated by Sanger sequencing.

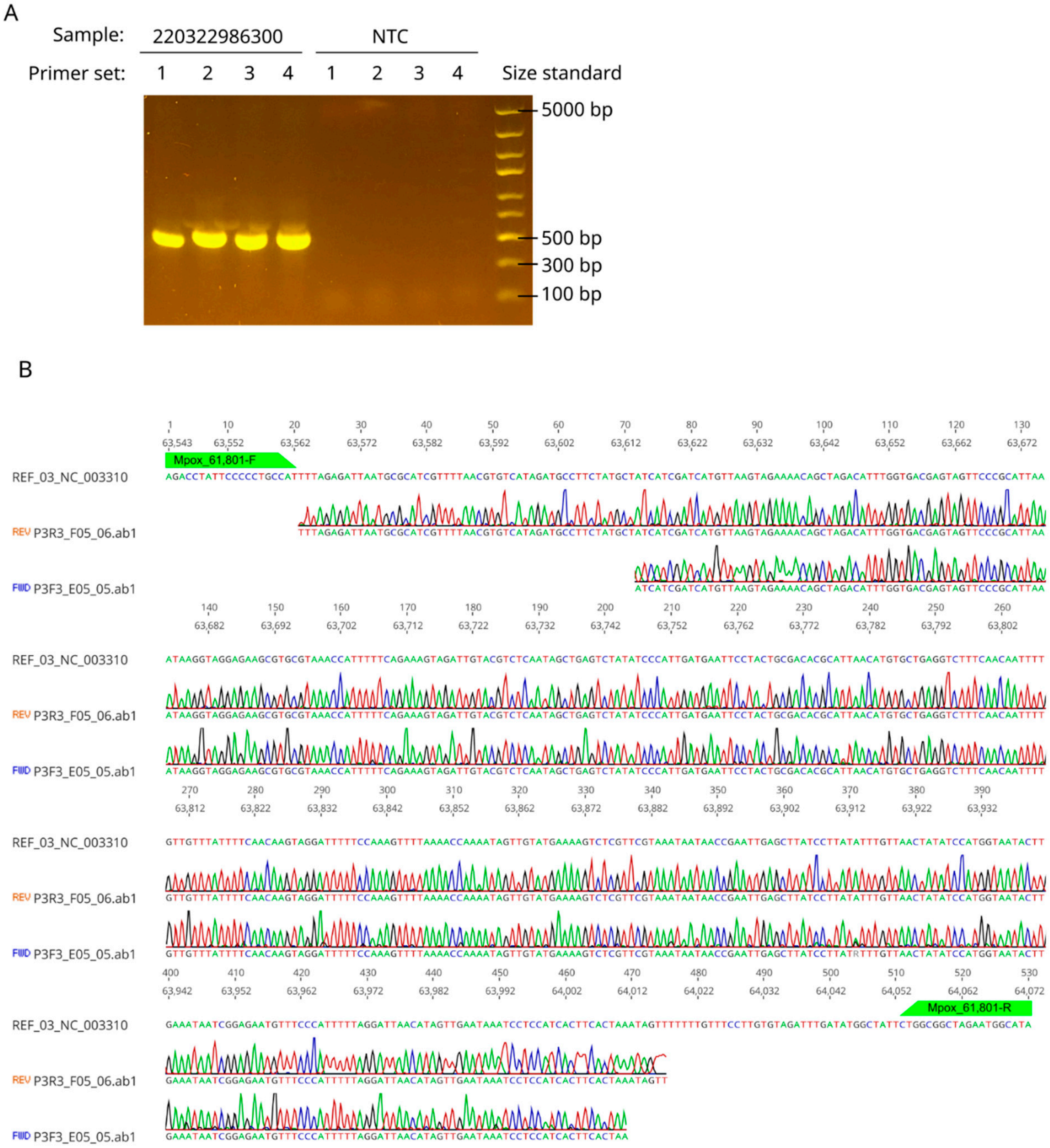

Figure S1. (A) Agarose gel electrophoresis of the PCR reaction for Sanger sequencing from sample 220322986300 and the NTC of sequencing regions MPX_SEQ_1, MPX_SEQ_2, MPX_SEQ_3, and MPX_SEQ_4. Alignment of the sequences generated by Sanger sequencing for the MPX_SEQ_3 region with the NC_003310 reference. The sequence P3R3_F05_06.ab1 corresponds to the reverse strand generated by synthesis from the primer Mpox_61,801-R. The sequence P3F3_E05_05.ab1 corresponds to the forward strand generated by synthesis from the primer Mpox_61,801-F. The positions relative to the amplicon (1–530) and the reference genome (63,543–64,072) are indicated.

3. Accuracy

The accuracy of our test was evaluated by comparison with the Central Public Health Laboratory of the Federal District (LACEN-DF), a reference laboratory of the Brazilian Ministry of Health for Monkeypox virus detection. The results for 30 samples are presented in Table S2.

Table S2. Comparability between our results and LACEN-DF results for 30 samples (15 with detected results and 15 with undetected results) showed 100% agreement.

N	Identification Number	Our Result	LACEN-DF Rwesult	Concordance Analysis
1	220322986300	DETECTED	DETECTED	TRUE
2	220326636400	DETECTED	DETECTED	TRUE
3	220326666500	DETECTED	DETECTED	TRUE
4	220327133900	NOT DETECTED	NOT DETECTED	TRUE
5	220327517200	DETECTED	DETECTED	TRUE
6	220329042600	DETECTED	DETECTED	TRUE
7	220329252500	DETECTED	DETECTED	TRUE
8	220330613300	DETECTED	DETECTED	TRUE
9	220332272503	NOT DETECTED	NOT DETECTED	TRUE
10	220340952201	NOT DETECTED	NOT DETECTED	TRUE
11	220341494400	NOT DETECTED	NOT DETECTED	TRUE
12	220342642100	DETECTED	DETECTED	TRUE
13	220344057104	DETECTED	DETECTED	TRUE
14	220347268200	NOT DETECTED	NOT DETECTED	TRUE
15	220347848000	DETECTED	DETECTED	TRUE
16	220351782500	NOT DETECTED	NOT DETECTED	TRUE
17	220353085700	NOT DETECTED	NOT DETECTED	TRUE
18	220353116800	NOT DETECTED	NOT DETECTED	TRUE
19	220352993500	DETECTED	DETECTED	TRUE
20	220353315102	NOT DETECTED	NOT DETECTED	TRUE
21	220354530300	DETECTED	DETECTED	TRUE
22	220354982102	NOT DETECTED	NOT DETECTED	TRUE
23	220356270200	DETECTED	DETECTED	TRUE
24	220356445600	DETECTED	DETECTED	TRUE
25	220356702300	DETECTED	DETECTED	TRUE
26	220356683200	NOT DETECTED	NOT DETECTED	TRUE
27	220357802100	NOT DETECTED	NOT DETECTED	TRUE
28	220358124100	NOT DETECTED	NOT DETECTED	TRUE
29	220358263500	NOT DETECTED	NOT DETECTED	TRUE
30	220360177300	NOT DETECTED	NOT DETECTED	TRUE
